# Initial Development and Psychometric Validation of the Self-Efficacy Scale for Informational Reading Strategies in Teacher Candidates

**DOI:** 10.3390/bs15081002

**Published:** 2025-07-23

**Authors:** Talha Göktentürk, Yiğit Omay, Ali Fuat Arıcı, Emre Yazıcı, Sevgen Özbaşı

**Affiliations:** 1 Department of Turkish Education, Faculty of Education, Yıldız Technical University, Davutpaşa Campus, 34220 Istanbul, Türkiye; 2 Department of Turkish Education, Faculty of Education, Başkent University, Bağlıca Campus, 06790 Ankara, Türkiye; sozbasi@baskent.edu.tr

**Keywords:** informal reading strategies, self-efficacy, scale development, teacher candidates, educational psychology

## Abstract

Assessing teacher candidates’ self-efficacy in using reading strategies is essential for understanding their academic development. This study developed and validated the Teacher Candidates’ Self-Efficacy Scale for Informational Reading Strategies (TCSES-IRS) using a mixed-methods sequential exploratory design. Initial qualitative data from interviews with 33 candidates and a literature review guided item generation. Lawshe’s method confirmed content validity. The scale was administered to 1176 teacher candidates. Exploratory (n = 496) and confirmatory factor analyses (n = 388) supported a five-factor structure—cognitive, note-taking, exploration and preparation, physical and process-based, and reflective and analytical strategies—explaining 63.71% of total variance, with acceptable fit indices (*χ*^2^/df = 2.64, CFI = 0.912, TLI = 0.900, RMSEA = 0.069). Internal consistency was high (α = 0.899 total; subscales α = 0.708–0.906). An additional sample of 294 participants was used for nomological network validation. Convergent validity was demonstrated by significant item-total correlations and strong factor loadings. Discriminant validity was evidenced by moderate inter-factor correlations. Criterion-related validity was confirmed via significant group differences and meaningful correlations with an external self-efficacy measure. The TCSES-IRS emerges as a psychometrically sound tool for assessing informational reading self-efficacy, supporting research and practice in educational psychology.

## 1. Introduction

Reading strategies refer to deliberate techniques readers employ to improve their comprehension, engagement, and retention of textual information (Arıcı, 2018; Habók et al., 2024). Within educational contexts, especially for teacher candidates, the use of informational reading strategies is particularly crucial, given the frequent need to comprehend, analyze, and critically evaluate complex informational texts encountered in academic coursework and professional training (Akkaya & Demirel, 2012). These strategies involve observable behaviors such as critical analysis, questioning, and information synthesis, ultimately fostering effective cognitive and behavioral engagement with informational texts (Özbay, 2011; Reis & Eckert, 2023). Proper use of these informational reading strategies empowers teacher candidates to question and evaluate texts critically, facilitating the construction of meaningful interpretations and strengthening their own comprehension in academic and professional contexts (R. Brown & Pressley, 2023). Teacher candidates aiming to enhance their comprehension and professional competencies rely on pre-planned, systematic strategies to deepen engagement and improve success in interpreting informational content (Baki, 2024; Karatay, 2023). These strategies can be categorized systematically into three distinct phases: pre-reading strategies, including goal setting and activating prior knowledge (Hoel et al., 2020; Omidire & Morgan, 2023); during-reading strategies, such as note-taking and summarizing to maintain focus and understanding of informational materials (Deane & Traga Philippakos, 2024); and post-reading strategies, which involve reflective evaluation and synthesis of the acquired knowledge (Akkaya & Demirel, 2012). This categorization enables a more focused examination of how teacher candidates apply informational reading strategies to access and use critical knowledge from texts.

Pre-reading strategies focus on preparing oneself to engage effectively with a text (Pellicer-Sánchez et al., 2021). These strategies help activate prior knowledge, set purposes for reading, and build expectations, enabling readers to create a mental framework for understanding the material (Hoel et al., 2020). Examples include skimming titles, headings, and illustrations to preview the content, as well as identifying critical vocabulary within a text to enhance one’s own familiarity with key concepts (Nosko, 2021). Techniques such as brainstorming, formulating questions, or using anticipation guides help readers establish connections between their prior knowledge and new information (Sinaga, 2024). In the context of teacher education, these strategies are essential for building preparatory schemas to understand complex informational texts, such as academic articles or instructional materials (Dixon & Oakhill, 2024; Fenty & Uliassi, 2018). These strategies make the initial engagement with a text more accessible and meaningful, especially when dealing with dense or unfamiliar material.

During-reading strategies support active engagement with the text by helping readers process and organize information (Tonks et al., 2021). These include techniques such as note-taking, underlining, or highlighting key ideas, which enhance retention and comprehension (Afflerbach et al., 2020). Readers may also employ strategies like questioning the content, summarizing sections, and using graphic organizers to identify main ideas and details (Aşıkcan et al., 2018). Contextual analysis, such as using clues within the text to infer the meaning of unknown words, is another valuable tool that fosters deeper interaction with the material (Moss et al., 2013). For teacher candidates, mastering these behaviors during informational reading—such as synthesizing curriculum guidelines or pedagogical theories—is crucial for academic performance and instructional preparedness (Karadağ, 2014). These strategies not only promote understanding but also encourage readers to critically analyze and evaluate the text as they read.

Post-reading strategies aim to consolidate comprehension and encourage reflection on the text (Akkaya & Demirel, 2012). Summarizing key points, generating questions, and engaging in discussions about the material are common techniques that reinforce understanding and promote knowledge retention (Nordin et al., 2013). These strategies also facilitate connections between the text and readers’ prior knowledge, helping them integrate new information into their existing frameworks (Serravallo, 2015). Tools like concept maps or chapter overviews allow readers to visually organize and review critical ideas, while activities such as writing reflections or completing practice exercises enable them to synthesize and apply what they have learned (Dennis et al., 2024; Mokhtari & Sheorey, 2008). Such reflective practices are particularly valuable for teacher candidates when engaging with informational content relevant to their field, as they reinforce pedagogical understanding and contribute to evidence-based decision-making (Crabtree-Groff, 2010; Slade et al., 2019).

By employing these three stages of reading strategies, readers can approach texts systematically, enhancing their ability to comprehend, retain, and critically engage with the material (Sermier Dessemontet et al., 2024). This tripartite framework becomes especially relevant in the context of teacher candidates, whose academic success and professional growth depend heavily on their ability to navigate informational texts effectively (Quinn & Paulick, 2022). A critical factor influencing the effective use of these strategies is self-efficacy, or individuals’ beliefs in their ability to plan and execute actions to achieve desired outcomes (Bandura, 1997; Gilbride, 2025). Teacher candidates’ self-efficacy in reading strategies not only shapes their confidence in navigating texts but also directly influences their observable behaviors, such as using goal-setting strategies during pre-reading or engaging in reflective practices post-reading (Karatay, 2023; S. Özdemir, 2018). Furthermore, developing self-efficacy in informational reading is essential for comprehending empirical research, policy documents, and theoretical frameworks—core components of teacher education (Starks, 2024). This is particularly significant for teacher candidates, who need to cultivate strong self-efficacy specifically in applying reading strategies to informational reading contexts, reflecting their frequent academic and professional reading tasks (Quinn & Paulick, 2022). Their self-efficacy perceptions directly influence their engagement with reading strategies and, consequently, their ability to foster these skills in classroom settings (Ryan & Hendry, 2023).

Teacher candidates must cultivate strong self-efficacy in the use of reading strategies, not only to enhance their own comprehension but also to serve as effective models for their future students (Dixon & Oakhill, 2024). Research indicates that teacher candidates who demonstrate proficiency in their personal use of reading strategies are better equipped to integrate these practices into instructional settings, effectively modeling and scaffolding reading behaviors (Quinn & Paulick, 2022). However, studies consistently highlight that many individuals enter teacher education programs with limited strategic reading habits and often lack sophisticated reading strategies, revealing a need for focused development in this area (González Ramírez & Pescara Vásquez, 2023; Orellana et al., 2024). By strengthening self-efficacy in applying reading strategies, teacher candidates can not only enhance their own reading proficiency but also facilitate meaningful reading experiences for their students, ultimately fostering improved academic outcomes and lifelong reading engagement (Lindström & Roberts, 2023). This is particularly pressing when considering the growing demand for evidence-based teaching practices, which require the ability to critically read and apply research findings—skills that are cultivated through strategic, self-efficacious engagement with informational texts (Khanshan & Yousefi, 2020; Ryan & Hendry, 2023).

Given the importance of reading strategies and their effective application, the role of self-efficacy emerges as a critical factor influencing not only the implementation of these strategies but also the overall development of teacher candidates. However, the limited availability of validated instruments to measure self-efficacy specifically in the context of informational reading strategies among teacher candidates poses a significant limitation in the field. Although general measures of reading self-efficacy exist (Arıcı, 2018; OECD, 2018), current instruments do not specifically address teacher candidates’ self-efficacy in applying reading strategies within informational contexts—a critical component of their academic and professional roles. This omission significantly limits our understanding of how teacher candidates’ personal perceptions of their reading capabilities influence their practical application of strategies and readiness to engage with informational texts in professional academic tasks. Moreover, without such focused tools, it becomes difficult to identify areas of instructional need or to design interventions that target the development of strategic informational reading for teacher candidates. Therefore, the current study focuses on the development of a scale intended for use with teacher candidates enrolled in undergraduate teacher education programs.

This study contributes to educational psychology by advancing the domain-specific understanding of self-efficacy through the development of a context-sensitive, theory-driven measurement tool. It operationalizes Bandura’s multidimensional model within a specialized literacy context—informational reading—thereby reinforcing the theoretical link between cognitive strategy use and motivational beliefs. From the perspective of teacher education, the scale provides educators and program designers with a validated instrument to assess and support teacher candidates’ strategic reading development, facilitating data-informed curriculum design, reflective teaching practices, and evidence-based interventions aimed at improving reading-related instructional readiness.

Addressing this gap is essential to better understand how teacher candidates perceive their capabilities in applying these strategies while reading and to provide them with tools that enhance their professional competence. The TCSES-IRS is specifically intended for use after teacher candidates have been admitted into teacher education programs, particularly during their coursework or practicum phases. Rather than serving as a tool for admissions screening, the scale is designed for formative and diagnostic purposes. It allows educators and program designers to monitor the development of informational reading self-efficacy, identify areas in which teacher candidates may lack confidence, and provide targeted support or instructional interventions accordingly. By doing so, we can better support teacher candidates’ strategic engagement with academic texts, which in turn informs their broader development as reflective professionals prepared to meet the demands of complex reading tasks within their academic and instructional environments. To ensure the rigor and validity of such tools, it is crucial to adhere to established guidelines in their development and evaluation.

To address this gap, this study developed and validated the Teacher Candidates’ Self-Efficacy Scale for Informational Reading Strategies (TCSES-IRS), a psychometric instrument guided by Bandura’s framework of self-efficacy (Bandura, 1997; Lawrent, 2024) and grounded in the domain-specific nature of informational reading within teacher education contexts (Lindström & Roberts, 2023; Orellana et al., 2024). The Standards for Educational and Psychological Testing, developed by the American Educational Research Association (AERA), the American Psychological Association (APA), and the National Council on Measurement in Education (NCME) (AERA et al., 2014), provide a framework for this test validity study. These standards establish guidelines for gathering multiple forms of validity evidence to justify score interpretations, ensuring a rigorous foundation for interpreting the scale’s results. Through this rigorous development and validation process, the TCSES-IRS emerges as a psychometrically sound instrument grounded in theory and rigorous standards, designed to advance research and inform practice in the crucial area of teacher candidates’ informational reading self-efficacy.

## 2. Theoretical Framework

### Bandura’s Theory of Self-Efficacy in the Context of Reading Strategies

Bandura’s theory of self-efficacy provides a critical framework for understanding how individuals develop confidence in their ability to perform specific tasks (Bandura, 2023). Self-efficacy refers to individuals’ beliefs in their capacity to organize and execute actions required to achieve desired outcomes (Bandura, 1997; Poluektova et al., 2023). These beliefs profoundly influence individuals’ motivation, effort, and resilience when encountering challenges (Saglam & Goktenturk, 2024; Shengyao et al., 2024). This concept is particularly relevant for assessing teacher candidates’ perceptions of their abilities to use reading strategies effectively, as these beliefs directly influence their engagement and persistence in applying such strategies (Ciampa et al., 2024). Bandura (2006) highlights the importance of clearly defining the behavioral domains of self-efficacy and emphasizes the need for domain-specific conceptualization to ensure theoretical coherence. Accordingly, informational reading represents a specific and important domain in teacher education, as teacher candidates frequently encounter complex informational texts requiring specialized strategies for successful comprehension and application.

Self-efficacy plays a critical role in shaping self-regulated behaviors and reflective practices in reading, particularly among teacher candidates (Chang & Bangsri, 2020; S. Li, 2023). By shaping individuals’ perceptions of their capabilities, self-efficacy acts as a motivational force, driving engagement with tasks, fostering persistence in the face of challenges, and promoting the adoption of behaviors essential for effective strategy use (Ciampa et al., 2024; el-Abd & Chaaban, 2021). Thus, enhancing self-efficacy among teacher candidates is essential not only for their personal academic development but also for their professional readiness to teach and model effective informational reading strategies to students (Ciampa et al., 2024; el-Abd & Chaaban, 2021).

According to Bandura, self-efficacy is shaped through four primary sources: mastery experiences, vicarious experiences, social persuasion, and emotional and physiological states (Bandura, 2006). These sources provide a theoretical foundation for understanding the construct of self-efficacy and serve as a basis for developing instruments that measure self-efficacy perceptions within specific contexts (Gale et al., 2021). Within the context of informational reading strategies, these four sources explain how teacher candidates build confidence across the sequential stages of reading: pre-reading, during-reading, and post-reading, thereby guiding their strategic approach to texts (Shehzad et al., 2020).

*Mastery experiences*, often regarded as the most influential source of self-efficacy, emphasize the importance of task success in building confidence (Lawrent, 2024). For teacher candidates, experiences of successfully applying pre-reading strategies, such as goal setting or previewing content, are central to strengthening their belief in their reading abilities (Peura et al., 2021). Similarly, positive *vicarious experiences*—observing peers or instructors effectively model strategies like note-taking or contextual analysis—help teacher candidates identify with successful practices, providing a foundation for their own confidence (el-Abd & Chaaban, 2021; Shehzad et al., 2020). The impact of vicarious experience is further magnified when individuals observe the success or failure of others who are perceived as similar to themselves, as this fosters stronger identification and perceived attainability of the behavior (Bandura, 1997; Poluektova et al., 2023).

*Social persuasion*, in the form of constructive feedback or encouragement, supports the development of self-efficacy by validating teacher candidates’ efforts and guiding their improvement in reading strategy use (Graham et al., 2020; Wyatt, 2022). Finally, *emotional and physiological states*, such as feelings of confidence or anxiety, influence perceptions of readiness to engage with reading tasks (Zarrinabadi et al., 2023). Managing these states, particularly during challenging reading phases, is essential for fostering a sense of efficacy. Therefore, Bandura’s (1997) framework, through its constituent sources, provides an essential theoretical grounding for investigating self-efficacy beliefs specific to teacher candidates’ use of informational reading strategies.

**The present study.** These four dimensions, as conceptualized by Bandura, highlight the multifaceted nature of self-efficacy (Poluektova et al., 2023). This multifaceted perspective is particularly valuable when examining informational reading strategies, as these strategies involve complex cognitive, motivational, and regulatory behaviors (Bandura, 2006; H. Li et al., 2022). They serve as a guiding framework for understanding the construct and developing tools that measure teacher candidates’ beliefs about their ability to use strategies effectively. In line with Bandura’s (2006) recommendations for domain-specific measurement, the current study seeks to develop an instrument tailored explicitly to informational reading contexts within teacher education.

By ensuring that the scale aligns with Bandura’s theoretical model, this study aims to develop and validate a reliable instrument capable of capturing teacher candidates’ self-efficacy perceptions across the pre-, during-, and post-reading phases. Thus, the investigation addresses a significant gap by providing a robust measurement tool specifically developed to reflect the unique reading demands encountered by teacher candidates. Drawing from qualitative interview data and insights from the literature, the research is designed to ensure that the tool reflects the nuanced dimensions of self-efficacy as they relate to reading strategies across different stages of the reading process.

To ensure methodological coherence, the first research question was addressed through the qualitative phase, which explored teacher candidates’ self-efficacy perceptions via semi-structured interviews and informed item generation. The second research question was investigated in the quantitative phase using exploratory and confirmatory factor analyses to test the scale’s construct validity and reliability. In alignment with this sequential mixed-methods design, the following research questions guided the study:

1. What are teacher candidates’ self-efficacy perceptions regarding their use of informational reading strategies across different reading phases (pre-, during-, and post-reading)?
2. To what extent is the newly developed scale valid and reliable for capturing teacher candidates’ self-efficacy perceptions and related behaviors regarding informational reading strategies?

**Figure 1:**
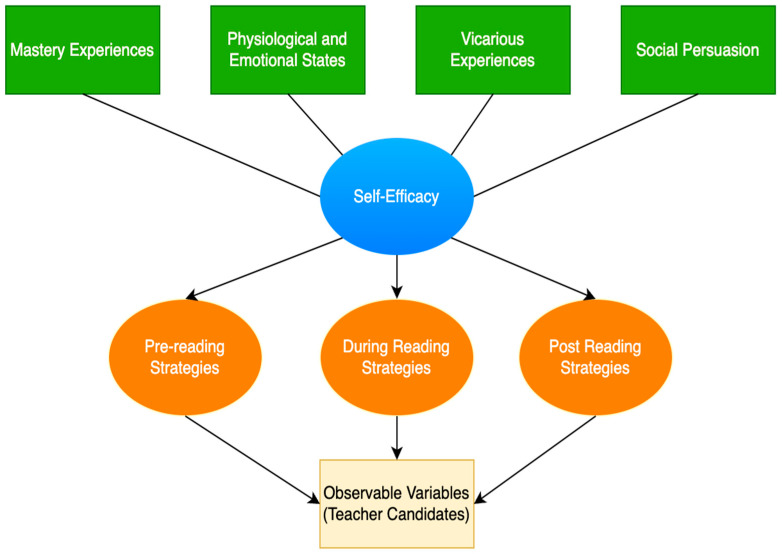
Conceptual framework guiding the scale development.

Addressing these questions contributes to educational psychology and teacher education by offering a rigorously validated scale that supports further research and instructional practices aimed at enhancing the informational reading competencies and professional preparation of teacher candidates. This theoretical alignment is visually represented in Figure 1.

## 3. Method

This study employed a mixed-methods sequential exploratory design (Creswell, 2012). Initially, a qualitative phase was conducted to explore teacher candidates’ perceptions and behaviors related to their use of informational reading strategies, followed by a quantitative phase focused on scale development and psychometric validation. This design was purposefully selected to first achieve an in-depth understanding of the phenomenon through qualitative exploration, ensuring subsequent quantitative scale items authentically represent participants’ experiences and contexts (Creswell & Poth, 2016).

### 3.1. Participants

#### 3.1.1. Qualitative Phase

Participants consisted of teacher candidates enrolled in teacher education programs at universities in Turkey. In the qualitative phase, 33 participants (36.36% women) were selected using purposive and snowball sampling to ensure data saturation and to capture a diverse range of perspectives across different academic years (Creswell, 2012; Hennink & Kaiser, 2022). Specifically, participants were drawn from first-year (n = 4), second-year (n = 7), third-year (n = 5), and fourth-year cohorts (n = 17). This process allowed for a representation of various teacher education programs, including Turkish Education, Elementary Mathematics Education, Social Sciences Education, Primary Teacher Education, Science Education, and Psychological Counseling and Guidance (Gill, 2020).

The sampling process began by identifying an initial participant and leveraging their network to recruit additional participants who met the inclusion criteria (Noy, 2008). Participants were required to be actively enrolled in a teacher education program, with no additional constraints such as GPA or prior self-efficacy assessment. Given the voluntary nature and snowball sampling approach, there was a possibility of self-selection bias, as individuals with higher confidence or greater engagement in reading strategies might have been more likely to participate. To partially mitigate this bias, participants were purposively selected from diverse academic years and departments, ensuring variation in experience and perceived confidence levels (Creswell, 2016). Data collection continued iteratively until thematic saturation was confirmed, with no new themes emerging from additional participants (Hennink & Kaiser, 2022). Table S2 in the Supplementary Materials provides detailed characteristics of the qualitative sample, including the participants’ universities, departments, and academic years.

Semi-structured interviews were conducted to explore participants’ self-efficacy perceptions and associated behaviors when engaging with informational texts, including academic articles, textbooks, and professional development materials commonly encountered in teacher education (Adeoye-Olatunde & Olenik, 2021; Kallio et al., 2016). This focus was important given that self-efficacy and strategy use may vary by genre, purpose, and task demands. The interview protocol (see Supplementary Materials, Table S3) was structured around pre-reading, during-reading, and post-reading phases and aligned with Bandura’s four sources of self-efficacy: mastery experiences, vicarious experiences, social persuasion, and physiological/affective states (Bandura, 2006; Ortlieb & Schatz, 2020).

#### 3.1.2. Quantitative Phase

In the quantitative phase, exploratory and confirmatory factor analyses were conducted using independent samples of teacher candidates who were actively enrolled in undergraduate teacher education programs at universities in Turkey. This approach was adopted to ensure methodological rigor and to minimize potential biases stemming from sample dependency (Lorenzo-Seva, 2021). An open invitation to participate was shared across diverse teacher education programs via a Google Form. Although the exact number of teacher candidates reached is not precisely known due to the use of informal class-level WhatsApp groups, it is estimated that approximately 1500 students were invited to participate. A total of 1176 students provided complete and valid responses, resulting in an estimated response rate of around 78%. These non-institutionally moderated groups serve as widely used communication platforms among teacher candidates, typically encompassing entire class cohorts. Participation was voluntary and anonymous, and each participant responded only once per data collection wave. As the Google Form enforced mandatory responses for each item, no missing data imputation procedures were necessary.

Following data collection, respondents were assigned unique numerical identifiers, and participants were randomly selected using SPSS 29 (IBM, 2021) to ensure unbiased and generalizable samples (Fraenkel et al., 2012). Initially, exploratory factor analysis (EFA) was conducted with 496 teacher candidates, yielding a stable and theoretically interpretable five-factor structure (Demir, 2020). Confirmatory factor analysis (CFA) was subsequently performed independently on a different sample of 388 teacher candidates (Supplementary Materials, Table S10), confirming the robustness and generalizability of the identified structure. Both samples exceeded the recommended participant-to-item ratio (10:1), ensuring adequate statistical power (Kyriazos, 2018). In addition, 294 new participants were recruited for testing nomological validity (Supplementary Materials, Table S11), including convergent, discriminant, and criterion-related validity analyses (AERA et al., 2014). Participants were selected using random sampling to enhance the generalizability of the findings to the broader population of teacher candidates (Fraenkel et al., 2012). The quantitative sample represented 14 different undergraduate programs, providing a diverse demographic profile and meeting recommended thresholds for rigorous psychometric analysis (Boateng et al., 2018; Rouquette & Falissard, 2011). To reduce redundancy and preserve readability, sample characteristics for the CFA and nomological validity samples are presented in the Supplementary Materials, while Table 1 in the main text focuses on the EFA sample, which forms the empirical basis for initial construct identification. Due to the nature of the open-access recruitment strategy via departmental mailing lists and student groups, an exact response rate could not be calculated. However, the final sample sizes exceeded recommended thresholds for factor analysis and validity testing (Kyriazos, 2018).

### 3.2. Data Analysis

#### 3.2.1. Qualitative Analysis

All interviews were analyzed using thematic content analysis within the framework of Bandura’s theoretical framework (Gilbride, 2025; Poluektova et al., 2023). NVivo 8 software was employed to facilitate the coding process, with the emergent categories grouped into overarching themes (QSR, 2008). The analysis process involved four iterative coding sessions among the researchers to ensure intercoder reliability and refine codes and themes (Miles et al., 2014). After achieving consensus, the final themes and codes were presented to an expert in Turkish education with experience in scale development for feedback. Following two rounds of consultation, adjustments were made to finalize the thematic structure (Braun & Clarke, 2006; Kerr et al., 2010). Subsequently, the qualitative data analysis informed the generation of the initial item pool, with detailed thematic findings provided in Supplementary Materials (Tables S4 and S5).

Ethical considerations were meticulously adhered to throughout the study. Verbal and written consent was obtained from all participants before the interviews, and the data were securely stored in encrypted folders by the researchers. Confidentiality was maintained by anonymizing participant responses (Creswell, 2016). The finalized thematic structure (Supplementary Materials, Tables S4 and S5) guided subsequent quantitative item generation.

#### 3.2.2. Quantitative Analysis

*Content validity*. The quantitative phase of the study aimed to validate the Teacher Candidates’ Self-Efficacy Scale for Informational Reading Strategies (TCSES-IRS). The scale, initially comprising 69 items, was designed to measure self-efficacy across three phases of reading—pre-reading, during-reading, and post-reading. The findings derived from the qualitative data explain self-efficacy behaviors emerging from the four sources identified by Bandura—mastery experiences, vicarious experiences, social persuasion, and emotional/physiological states—across the pre-reading, during-reading, and post-reading phases (Creswell, 2009). For example, items representing mastery experiences included statements such as, ‘I can identify the most important points in a complex text,’ reflecting teacher candidates’ confidence in task success. Based on these sources, the self-efficacy behaviors of teacher candidates recorded in relation to the reading processes were translated into scale items, shaping the final draft of the instrument (Creswell & Poth, 2016).

To provide evidence based on test content in accordance with the standards (AERA et al., 2014), the initial draft of the scale was reviewed by nine experts with relevant expertise (DeVellis & Thorpe, 2021). Four experts were Turkish education specialists with experience in scale development, while five were educational scientists with expertise in self-efficacy and psychometrics. Each expert evaluated the relevance of the items using Lawshe’s content validity ratio (CVR) method (Lawshe, 1975). Based on the panel size, the minimum acceptable CVR threshold was set at 0.78 (Supplementary Materials, Table S6). The review process refined ambiguous items and ensured alignment with theoretical constructs, resulting in clearer and more focused items. Items that did not meet this standard were revised or eliminated, resulting in the removal of 20 items. The refined scale included 49 items, which were retained for subsequent analyses (L. A. Clark & Watson, 2016).

*Face validity*. Face validity was ensured through a pilot application of the scale to a small group of teacher candidates (Connell et al., 2018). Participants provided feedback on the clarity, relevance, and comprehensibility of the items. Specific changes included simplifying complex wording and adjusting terminology to ensure alignment with participants’ experiences. Based on their suggestions, minor adjustments were made to enhance readability and ensure the items were contextually appropriate for the target population (Sato & Ikeda, 2015).

*Construct validity*. To guide item generation, a thematic analysis was conducted on qualitative data gathered from teacher candidates, who described their self-efficacious reading behaviors (Braun & Clarke, 2024). These behaviors were initially organized within a temporal framework—pre-reading, during-reading, and post-reading—to ensure coverage of the full scope of strategy use across different phases of the reading process (Karatay, 2023). This time-based structure was not intended as a multilevel construct but rather as a tool to capture a comprehensive range of contextualized experiences, consistent with the notion that self-efficacy beliefs may emerge from perceptions anchored in past, present, or future events.

Thematic coding was guided by Bandura’s four sources of self-efficacy—mastery experiences, vicarious experiences, social persuasion, and physiological/affective states—(Lawrent, 2024) and independently reviewed by an educational sciences expert to confirm the coherence of the thematic groupings (Miles et al., 2014). The five identified factors aligned meaningfully with these theoretical dimensions: cognitive strategies reflected both mastery and vicarious experiences, note-taking strategies were associated with mastery experiences and social persuasion, and exploration and preparation strategies aligned with vicarious experiences and social persuasion. Physical and process-based strategies corresponded to physiological and affective states, while reflective and analytical strategies encompassed mastery experiences and physiological/affective states. Representing distinct yet interconnected dimensions of reading self-efficacy, these five factors were incorporated into SPSS 29 as an initial five-factor model for exploratory analysis (IBM, 2021). This approach ensured methodological rigor, theoretical grounding in Bandura’s framework, and reliability in interpreting the relationships between self-efficacy constructs and observed strategies.

Following the standards’ guidelines (AERA et al., 2014), construct validity was assessed through both exploratory (Reio & Shuck, 2015) and confirmatory (T. A. Brown, 2023), factor analyses conducted on the full dataset of 884 participants. Exploratory factor analysis (EFA; n = 496) was performed using principal component analysis with varimax rotation to identify the underlying factor structure (Loewen & Gonulal, 2015). Although PCA is often viewed as a data reduction technique rather than a latent variable method, it was chosen for its robustness against normality violations and ability to maximize explained variance while preserving theoretical clarity (Jolliffe & Cadima, 2016). Principal Axis Factoring (PAF), which extracts only common variance (Howard & O’Sullivan, 2024) and is more sensitive to sample sizes (de Winter & Dodou, 2012), was not used because the goal was to establish an empirically stable and practically interpretable factor structure rather than infer latent constructs. PCA was preferred as it incorporates all available variance, making it particularly suitable for exploratory scale development in self-efficacy research (Demir, 2020; Loewen & Gonulal, 2015). Unlike Maximum Likelihood (ML) estimation, which assumes multivariate normality and is commonly used in factor extraction for exploratory analyses when latent variable modeling is intended (Y. Chen et al., 2019), PCA offers a practical approach for identifying distinct, interpretable factor structures in exploratory scale development (Loewen & Gonulal, 2015). PCA is widely used in educational psychology research for efficiently deriving meaningful dimensions in self-efficacy studies (Dembereldorj et al., 2023; Kurudayıoğlu et al., 2021). Varimax rotation was selected over oblique rotation (e.g., Promax) to enhance interpretability by producing distinct, minimally correlated factor structures (Sass & Schmitt, 2010). While self-efficacy dimensions are theoretically interrelated, the objective was to identify clear, actionable dimensions aligned with Bandura’s self-efficacy framework (Warner & French, 2020).

Items with low factor loadings (below 0.40) or significant cross-loadings (factor loadings on more than one factor with a difference of less than 0.10) were removed (Boateng et al., 2018; Howard, 2016). After this process, 24 items remained, forming the final version of the scale. Confirmatory factor analysis (CFA; n = 388) was conducted using R’s *lavaan* package with maximum likelihood (ML) estimation to validate the factor structure identified through EFA (Rosseel, 2012; Xia & Yang, 2019). The model’s adequacy was assessed using key fit indices, including the chi-square statistic (*χ*^2^/df ≤ 3 for acceptable fit), comparative fit index (CFI ≥ 0.90 for acceptable fit, ≥0.95 for excellent fit), Tucker–Lewis index (TLI ≥ 0.90 for acceptable fit, ≥0.95 for excellent fit), root mean square error of approximation (RMSEA ≤ 0.08 for acceptable fit, ≤0.05 for excellent fit), and standardized root mean square residual (SRMR ≤ 0.08 for acceptable fit, ≤0.05 for excellent fit) (D. A. Clark & Bowles, 2018; Finch, 2020). Exploratory factor analysis (EFA; n = 496) and confirmatory factor analysis (CFA; n = 388) were conducted on independent samples, exceeding the recommended threshold of 10 participants per item to ensure sufficient statistical power for factor analysis (Kyriazos, 2018).

During the item development phase, all qualitative codes—including less frequent or narrowly contextual behaviors such as *post-reading discussions*—were initially represented in the draft item pool. However, during exploratory factor analysis, items corresponding to certain qualitative themes demonstrated low factor loadings or significant cross-loadings across multiple factors, failing to meet statistical thresholds for retention (Boateng et al., 2018; Howard, 2016). Specifically, themes like *post-reading discussions* were excluded because their associated items did not form a coherent latent structure or consistently align with the broader self-efficacy construct. These decisions were grounded in both statistical criteria and theoretical relevance to the targeted construct.

*Reliability*. Reliability was assessed through Cronbach’s alpha coefficients for the overall scale and its subdimensions (Kılıç, 2016; Streiner, 2003). The coefficients indicated high internal consistency, with all values exceeding the threshold of 0.70 (Demir, 2020). Correlations between item scores and total scores were also examined, verifying that each item contributed meaningfully to its respective factor (L. A. Clark & Watson, 2016; DeVellis & Thorpe, 2021). To further validate the scale, item, factor, and total scores were compared between the upper 27% and lower 27% of participants (Boateng et al., 2018). Significant differences across these comparisons indicated that the scale effectively distinguishes varying levels of self-efficacy among teacher candidates.

*Convergent and discriminant validity*. Following factor extraction, Pearson correlation analyses were conducted to examine the relationships among the extracted factor scores as part of construct validity assessment (H. F. Özdemir et al., 2019). This two-step approach—using PCA for initial factor extraction and correlation analysis for construct validity—maintains methodological rigor while aligning with theoretical expectations. Item-total correlations and factor loadings confirmed convergent validity, while inter-factor correlations supported discriminant validity. These findings aligned with the theoretical assumption that self-efficacy dimensions are related but distinct (L. A. Clark & Watson, 2016; DeVellis & Thorpe, 2021). To support convergent validity, a correlation analysis was conducted between the total score of the developed scale and the perception of competence in reading index (ST161) adapted from PISA 2018 (OECD, 2019). For discriminant validity, an additional Pearson correlation was performed with the mathematics self-efficacy index (ST290) derived from PISA 2022 to test the distinction between informational reading and unrelated domains (OECD, 2024).

*Criterion-related validity*. To support criterion-related validity, five informational reading items (Items 1, 3, 15, 23, and 25) from the Turkish Public Personnel Selection Examination (KPSS, 2021) were administered (ÖSYM, 2021). Participants’ total scores on these items were used as the dependent variable in a linear regression model, with self-efficacy scores entered as predictors to examine the scale’s ability to forecast actual informational reading performance (AERA et al., 2014). This approach allowed for an authentic, performance-based evaluation of predictive validity.

## 4. Results

### 4.1. Qualitative Results

Thematic analysis of the semi-structured interviews revealed four primary themes aligned with Bandura’s theoretical framework: (1) mastery experiences, where participants described how past success with informational texts increased their confidence (e.g., “When I summarized a research article well, I felt more confident the next time.”); (2) vicarious experiences, as candidates gained confidence by observing peers using strategies effectively (e.g., “I saw my classmate using note-taking templates, so I tried it too.”); (3) social persuasion, including encouragement from instructors (e.g., “My advisor said I was improving a lot in how I read academic texts.”); and (4) emotional and physiological states, where stress or fatigue diminished perceived self-efficacy (e.g., “When I’m overwhelmed, I don’t believe I can understand the material.”). These themes informed the generation of item pools and the structuring of scale dimensions. Full thematic breakdowns and representative excerpts are provided in Supplementary Materials (Tables S4 and S5).

### 4.2. Quantitative Results

#### 4.2.1. Construct Validity

##### Exploratory Factor Analysis: Identifying the Factor Structure of the Scale

The exploratory factor analysis (EFA) aimed to uncover the underlying structure of the Teacher Candidates’ Self-Efficacy Scale for Informational Reading Strategies (TCSES-IRS) Initially, the scale comprised 49 items, which were evaluated for retention based on factor loadings, communalities, and conceptual coherence. The Kaiser-Meyer-Olkin (KMO) measure of sampling adequacy was 0.896, indicating suitability for factor analysis, and Bartlett’s Test of Sphericity was significant (*χ*^2^ = 6112.03, *p* < 0.001), confirming that the dataset was factorable. Principal component analysis with varimax rotation revealed a five-factor structure that explained 63.71% of the total variance.

Items with factor loadings below 0.40 or significant cross-loadings (≤0.10 across multiple factors) were removed, resulting in a final pool of 24 items. Table 2 presents the factor loadings for each item, and Table 3 shows the cumulative variance explained by each factor. The factors were named based on theoretical alignment with Bandura’s sources of self-efficacy and the content of the items.

*Cognitive Strategies* (Factor 1). This factor represents cognitive processes employed by teacher candidates to enhance reading comprehension and information retention. It includes strategies such as critically analyzing text content, connecting new ideas to prior knowledge, and drawing logical inferences. The factor consists of nine items.

*Note-Taking Strategies* (Factor 2). This factor encompasses practical methods that teacher candidates employ to record and recall important information during reading. It includes behaviors such as underlining key concepts, systematically reviewing personal notes, and annotating texts for later reference. This factor comprises four items.

*Exploration and Preparation Strategies* (Factor 3). This factor relates to proactive steps teacher candidates undertake before engaging with a text to establish contextual comprehension and facilitate effective reading. It covers strategies such as researching the author, examining historical and cultural contexts of texts, and pinpointing key themes. This factor includes four items.

*Physical and Process-Based Strategies* (Factor 4). This factor captures the physical and environmental adjustments made by teacher candidates to optimize their reading experience. It includes behaviors such as selecting appropriate lighting, adjusting posture, and regulating the reading environment. This factor consists of four items.

*Reflective and Analytical Strategies* (Factor 5). This factor pertains to reflective and critical evaluation activities that teacher candidates practice following the reading process. It involves critically assessing the arguments presented, analyzing thematic undercurrents of the text, and articulating insights through discussions. This factor contains three items.

The scree plot (see Supplementary Materials, Figure S1) supported the retention of a five-factor model. The eigenvalues of the first five factors were 7.90, 2.49, 1.94, 1.68, and 1.28, all exceeding the commonly accepted threshold of 1.00, with a distinct “elbow” observed after the fifth factor, indicating the optimal point for factor retention. Table 3 provides a detailed summary of the eigenvalues, the percentage of variance explained by each factor, and the cumulative variance accounted for by the five-factor solution.

##### Confirmatory Factor Analysis

**Figure 2:**
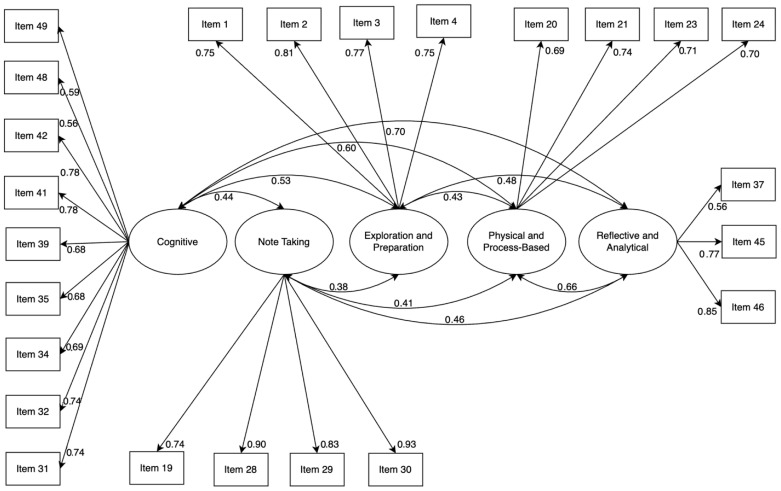
The path diagram of confirmatory factor analysis.

Confirmatory factor analysis (CFA) was performed using R’s lavaan to validate the factor structure identified through EFA. The CFA was conducted with 388 participants. Detailed demographic characteristics of this expanded sample are provided in the Supplementary Materials (Table S10). The CFA results with the larger sample demonstrated an adequate fit to the data: *χ*^2^/df = 2.64, Comparative Fit Index (CFI) = 0.912, Tucker–Lewis Index (TLI) = 0.90, root mean square error of approximation (RMSEA) = 0.069, and standardized root mean square residual (SRMR) = 0.056. These values meet commonly accepted thresholds for model fit (D. A. Clark & Bowles, 2018; Finch, 2020). The CFA confirmed the five-factor structure, validating the theoretical and empirical robustness of the scale. Figure 2 illustrates the standardized factor loadings and error variances for the final model.

#### 4.2.2. Reliability

##### Cronbach’s Alpha Values

The reliability of the scale was assessed using Cronbach’s Alpha coefficients. The total scale demonstrated excellent internal consistency (α = 0.899). Reliability values for each factor ranged from 0.708 to 0.906, as detailed in Table 4, indicating high reliability for all subscales. The reliability findings align with existing standards for psychological and educational measurement tools (George & Mallery, 2011). These results affirm the consistency and dependability of the scale for measuring teacher candidates’ self-efficacy in using reading strategies.

##### Item and Factor Discrimination: Independent-Sample *t*-Test Results

Independent-sample *t*-tests were conducted between the upper 27% and lower 27% of participants based on total scores to evaluate the discriminative power of the scale. All items and factors exhibited significant differences (*p* < 0.001), confirming their ability to distinguish between teacher candidates with varying levels of self-efficacy. The detailed statistics of the *t*-test results are provided in the Supplementary Materials (Tables S8 and S9), highlighting the significant distinctions across items and factors. These findings underscore the validity of the scale in measuring differences in self-efficacy perceptions.

##### Inter-Factor Relationships: Correlation Analysis

To evaluate the relationships among factors and their alignment with the total scale score, Pearson correlation analysis was conducted. The results indicated weak to strong positive correlations across all factors, with values ranging from r = 0.234 to r = 0.532, suggesting that the factors, while distinct, are interrelated and collectively contribute to the overarching construct of self-efficacy.

The detailed correlation matrix is presented in Table 5, illustrating the relationships between individual factors and the total scale score. Notably, cognitive strategies exhibited significant positive correlations with all other factors, with the strongest correlation observed with reflective and analytical strategies (r = 0.532). Similarly, note-taking strategies showed moderate correlations with other factors, such as physical and process-based strategies (r = 0.347). Among all relationships, the strongest overall correlation was found between cognitive strategies and the total scale score (r = 0.814), followed by reflective and analytical strategies (r = 0.691), and note-taking strategies (r = 0.683).

These findings provide robust evidence of the scale’s internal consistency and validate its theoretical structure by demonstrating the interdependence and coherence of the identified factors. They highlight the extent to which each factor contributes uniquely yet synergistically to the construct of teacher candidates’ self-efficacy in reading strategies.

#### 4.2.3. Convergent and Discriminant Validity

To evaluate convergent validity, Pearson’s correlation analysis was conducted between the total score of the TCSES-IRS and the perception of competence in reading index (ST161), adapted from PISA 2018. The analysis revealed a significant and positive correlation (*r* = 0.215, *p* < 0.001), indicating that teacher candidates with higher self-efficacy in informational reading strategies also tended to perceive themselves as more competent readers. For discriminant validity, the TCSES-IRS total score was correlated with the mathematics self-efficacy index (ST290) from PISA 2022. The resulting correlation was weak and non-significant (*r* = 0.052, *p* = 0.367), supporting the assumption that the construct of informational reading self-efficacy is distinct from self-efficacy beliefs in unrelated academic domains such as mathematics. These results provide empirical support for the scale’s convergent and discriminant validity.

#### 4.2.4. Criterion-Related Validity

To evaluate criterion-related validity, a simple linear regression analysis was conducted to examine the predictive power of the TCSES-IRS for actual informational reading performance. Participants’ scores on five items from the Turkish Public Personnel Selection Examination (KPSS, 2021) served as the criterion variable. The analysis revealed that informational reading self-efficacy significantly predicted reading performance (β = 0.14, *p* = 0.020), providing empirical support for the scale’s predictive validity in an authentic assessment context.

## 5. Discussion

The present study aimed to develop and validate the Teacher Candidates’ Self-Efficacy Scale for Informational Reading Strategies (TCSES-IRS), grounded in Bandura’s self-efficacy theory and informed by qualitative and quantitative analyses. The study utilized a mixed-methods approach, beginning with a qualitative exploration of teacher candidates’ reading strategies and culminating in a five-factor scale validated through exploratory and confirmatory factor analyses (Creswell, 2012). To further support the validity argument, convergent, discriminant, and criterion-related evidence was examined, reinforcing the scale’s external alignment with related constructs. Correlation analyses confirmed satisfactory convergent, discriminant, and criterion-related validity by showing that informational reading self-efficacy was positively associated with general reading self-efficacy, showed no significant relationship with mathematics self-efficacy, and modestly predicted performance on a national reading comprehension test (KPSS, 2021). These findings collectively affirm the nomological validity of the TCSES-IRS, establishing its position within a broader network of theoretically related constructs (AERA et al., 2014), and showcasing its applicability in educational assessment and behavioral science research. Thus, a scale supported by multiple sources of validity evidence was developed, demonstrating strong internal consistency and theoretical alignment, while recognizing that validity is an ongoing, context-specific process (DeVellis & Thorpe, 2021).

The qualitative phase was instrumental in identifying behaviors and strategies that teacher candidates employ across pre-reading, during-reading, and post-reading phases (Arıcı, 2018; Karatay, 2023). Themes and categories derived from the qualitative data reflected all four sources of self-efficacy described by Bandura: mastery experiences, vicarious experiences, social persuasion, and emotional/physiological states (Bandura, 2023). For instance, strategies such as “analyzing the author’s perspective” and “researching the historical context of the text” align closely with mastery experiences (Bandura, 2023), while strategies like “discussing the text with peers” embody vicarious experiences and social persuasion (Gilbride, 2025).

During the quantitative phase, the exploratory factor analysis revealed five distinct factors: (1) cognitive strategies, (2) note-taking strategies, (3) exploration and preparation strategies, (4) physical and process-based strategies, and (5) reflective and analytical strategies. While most of these factors emerged directly from the qualitative findings, some qualitative categories, such as “post-reading discussions”, were not strongly represented in the final factor structure. This discrepancy may indicate that certain strategies, while valuable in practice, do not significantly contribute to the latent construct of self-efficacy in the context of reading strategies (Gorsuch, 2003, 2014). However, it is worth noting that these categories may emerge in future self-efficacy studies targeting different populations, especially considering their relevance in the existing literature (Arıcı, 2018; Karatay, 2023).

Although the five-factor structure was not explicitly designed as a hierarchical model based on pre-, during-, and post-reading stages (Arıcı, 2018; Karatay, 2023), the identified factors align closely with these phases in practice. Exploration and preparation strategies predominantly reflect pre-reading behaviors, such as researching a text’s context and purpose. During-reading practices are represented by note-taking strategies and physical/process-based strategies, including annotating texts and managing reading conditions (e.g., focus, environment). Conversely, cognitive strategies and reflective/analytical strategies largely correspond to post-reading processes, such as interpreting, synthesizing, and critically evaluating content. By mapping specific self-efficacy dimensions to these phases, the TCSES-IRS provides a practical framework for both researchers and educators to diagnose and support teacher candidates’ strategic reading confidence throughout the full reading cycle.

The results highlight the integral role of Bandura’s four sources of self-efficacy in the scale’s factor structure (Bandura, 1997; Poluektova et al., 2023). Cognitive strategies and note-taking strategies were found to strongly reflect mastery experiences, as they emphasize independent problem-solving and active engagement with texts (Zhang, 2018). Reflective and analytical strategies align with emotional/physiological states, as they involve critical engagement and post-reading reflection, which may enhance confidence in managing challenging texts (Zaccoletti et al., 2023). Additionally, exploration and preparation strategies and note-taking strategies represent vicarious experiences and social persuasion by fostering behaviors like adopting modeled strategies and seeking peer feedback (Koponen et al., 2021; Pei & Bao, 2020). Improved self-efficacy in these strategies not only enhances academic success but also fosters confidence, persistence, and adaptability, which are essential for lifelong learning and professional growth (Ciampa et al., 2024; Muche et al., 2024).

Interestingly, physical and process-based strategies introduce a novel dimension to self-efficacy in reading strategies, focusing on environmental and physical adjustments to facilitate optimal reading conditions (Göktentürk, 2021; Sağlam et al., 2023). This emphasizes the interplay between self-regulatory behaviors, cognitive processes, and external environmental contexts, showcasing the behavioral science implications of these findings. This finding aligns closely with Bandura’s concept of Triadic Reciprocal Determinism, which emphasizes the dynamic interaction between personal factors, behaviors, and environmental influences (Woodcock & Tournaki, 2023). Specifically, the emergence of physical and process-based strategies illustrates how environmental conditions, such as the physical reading context, directly influence behaviors (e.g., adjusting posture, managing distractions), and subsequently, how these behaviors reinforce teacher candidates’ personal beliefs about their capabilities (self-efficacy) (McCarthy & Newcomb, 2014; Revilla, 2023). By explicitly integrating Bandura’s reciprocal determinism, this scale offers both a theoretically coherent understanding of reading self-efficacy and practical insights for teacher educators—emphasizing how strategic confidence translates into improved comprehension, critical thinking, and academic achievement (Gilbride, 2025).

Beyond its methodological contributions, this study also advances theoretical discourse in educational psychology and offers practical applications for teacher education. By operationalizing Bandura’s model of self-efficacy in the specific domain of informational reading, the TCSES-IRS provides a framework for understanding how cognitive, emotional, and contextual factors shape teacher candidates’ strategic reading behaviors. For educational psychology, the scale offers a validated tool to examine motivation-related constructs within authentic learning tasks. In teacher education, the instrument enables educators to diagnose areas of strength and need in reading strategy use, informing data-driven instructional design, targeted feedback, and professional development initiatives. Through this dual contribution, the study bridges conceptual theory and applied educational practice.

## 6. Conclusions

The Teacher Candidates’ Self-Efficacy Scale for Informational Reading Strategies provides a novel, empirically grounded tool to assess the multifaceted dimensions of reading self-efficacy in teacher education. This scale bridges theoretical constructs and practical needs, offering valuable insights for both research and educational practice. Its implications extend beyond educational contexts, contributing to broader behavioral science discussions on how self-efficacy impacts cognitive and motivational processes. Moreover, its explicit focus on informational reading contexts aligns closely with teacher candidates’ academic and professional reading demands, supporting their development as strategic, self-efficacious readers in educational settings.

TCSES-IRS not only fills a gap in educational measurement but also contributes to behavioral science methodology by offering a tool supported by multiple sources of validity evidence for assessing a multifaceted psychological construct. This scale provides researchers with a robust framework to investigate the cognitive, emotional, and environmental dimensions of self-efficacy (Wyatt, 2022). By representing subdimensions such as reflective strategies and exploration behaviors, the scale enables a deeper exploration of how these factors interact to shape behavior in educational and professional settings. Thus, this study contributes to behavioral sciences by providing a deeper understanding of self-efficacy as a multifaceted construct that combines cognitive, emotional, and environmental dimensions. In alignment with standards (AERA et al., 2014), this validated scale provides evidence supporting the interpretation of scores for assessing self-efficacy in reading strategies among teacher candidates. The validity evidence gathered aligns with the standards’ framework, incorporating content, construct, and criterion-related validity. Beyond its application in education, the scale serves as a valuable tool for examining the role of self-efficacy in shaping reading behaviors across different contexts. It also contributes to broader theoretical and methodological discussions in behavioral sciences by offering insights into how self-efficacy mechanisms interact with learning strategies. Additionally, this scale holds potential for informing targeted interventions aimed at enhancing motivation (Schunk & DiBenedetto, 2021), adaptability (C. Chen et al., 2023), and resilience (S. Li, 2023) across various professional and personal domains.

## 7. Limitations, Implications, and Future Research

While this study offers valuable insights, several limitations should be acknowledged to ensure a comprehensive interpretation of the findings. According to the Standards for Educational and Psychological Testing (AERA et al., 2014), no single study can provide exhaustive validity evidence. Although substantial validity evidence was gathered in the present research, additional forms of evidence—such as response processes and longitudinal predictive validity—are necessary to further strengthen the validity argument.

First, the exclusion of some qualitative categories in the final factor structure may indicate the need for additional exploration of their relevance. Future studies could employ longitudinal qualitative designs or mixed-method approaches to investigate the dynamic relationship between these omitted strategies and self-efficacy in different contexts (De Lisle, 2011; Gale et al., 2021).

Secondly, while the purposive and snowball sampling methods facilitated access to diverse participants in the qualitative phase, they may have introduced self-selection bias (Creswell, 2016). Specifically, participants who volunteered to be interviewed might have had a stronger sense of self-efficacy or greater familiarity with reading strategies, potentially influencing the range of behaviors reported. Future research should incorporate randomized or stratified qualitative sampling techniques to enhance the representativeness and transferability of qualitative themes.

Thirdly, although an increased sample size improved the model fit indices in the confirmatory factor analysis, additional validation across diverse populations is recommended to establish broader generalizability (T. A. Brown, 2015, 2023). Such validation should extend to diverse populations, including in-service teachers, different age groups (e.g., high school students, adult learners), and other educational stakeholders across various cultural contexts. While the current study focused on Turkish teacher candidates enrolled in undergraduate programs, additional research is needed to examine the scale’s applicability in different educational systems and professional stages. This will help clarify the scale’s broader utility and generalizability. Although measurement invariance testing was not a primary aim of this study, it represents a critical direction for future research. The current study employed a simple random sampling approach, focusing on developing a scale with generalizable validity evidence across the full teacher candidate population rather than stratified subgroups. As a result, investigating measurement invariance across demographic categories was considered outside the scope of the validation process. Future studies using stratified or multistage sampling with adequately powered subgroups are encouraged to explore the cross-group equivalence of the scale (Bulut, 2020; Erkuş, 2016)

Fourthly, the study relied on self-reported data, assuming participants’ honesty and accuracy (Brutus et al., 2013). While anonymity and confidentiality were assured, future research could incorporate supplementary data collection methods, such as observational studies or peer evaluations, to validate self-reported measures of self-efficacy (Creswell, 2012). Additionally, future research should also include measurement invariance studies to enhance the cross-context validity of the scale (Bulut, 2020; Erkuş, 2016).

Lastly, although our five-factor structure emerged through both theory-informed item development and empirical analysis, the possibility of a higher-order factor model—wherein subdimensions load onto broader latent constructs representing pre-, during-, and post-reading stages—was not tested in the current study. Future research should explore the applicability of second-order or bifactor models to examine whether this theoretical organization yields improved model fit or explanatory power.

From a practical perspective, this study provides significant opportunities for educators and policymakers. The validated five-factor scale can be employed as a diagnostic tool in teacher education programs to identify areas where teacher candidates may require additional support or training in employing reading strategies (Kelley & Zygouris-Coe, 2006). Educators can use these findings to design targeted interventions, such as workshops or mentoring programs, that emphasize enhancing less-developed strategies like reflective or goal-oriented reading practices. Policymakers might consider integrating the scale into teacher certification processes or continuous professional development programs, emphasizing self-efficacy as a foundational aspect of instructional quality.

Although the present study did not establish formal cutoff points for high or low self-efficacy levels, the TCSES-IRS enables dimensional interpretation of both total and subscale scores, where higher scores reflect stronger perceived competence in strategy use. For future implementation, researchers and educators may consider deriving norm-referenced benchmarks or percentile-based categorizations to guide score interpretation. This would enable the identification of teacher candidates who may benefit from targeted instructional interventions or further pedagogical support.

For researchers, the study opens new paths for investigating the interplay between reading self-efficacy and broader cognitive and behavioral outcomes, such as motivation, resilience, and adaptability (Gómez & Rivas, 2022). Future research may pose targeted questions using the TCSES-IRS, such as: How does reading self-efficacy develop across different semesters or practicum experiences in teacher education programs? Which instructional interventions are most effective in enhancing specific self-efficacy dimensions like reflective or note-taking strategies? How does informational reading self-efficacy predict instructional decision-making and professional reading behaviors in real-world teaching contexts? Future studies employing longitudinal designs can explore how changes in reading self-efficacy impact these outcomes over time, thereby contributing to theoretical advances in behavioral sciences. Tracking teacher candidates from pre-service through in-service stages may offer insights into the developmental trajectory of self-efficacy and its implications for literacy pedagogy. Additionally, interdisciplinary research should examine interactions between this scale and related constructs, including emotional intelligence, metacognition, and social-emotional learning, across diverse populations. This way, this research not only lays a strong foundation for practical applications in educational and policy contexts but also enriches the broader understanding of self-efficacy as a critical driver of meaningful change in both academic and professional domains.

## Figures and Tables

**Table 1:** Sample characteristics of quantitative stage for exploratory factor analysis.

Variable	n = 496 (%)
Sex	
Man	104 (20.97%)
Woman	392 (79.03%)
Undergraduate program	
Computer Education and Instructional Technology	6 (1.22%)
English Language Teaching	15 (3.04%)
Geography Education	1 (0.2%)
Elementary Mathematics Education	27 (5.48%)
Physical Education Teaching	4 (0.81%)
Preschool Education	37 (7.51%)
Primary Teacher Education	62 (12.58%)
Psychological Counseling and Guidance	24 (4.87%)
Science Education	35 (7.1%)
Social Studies Education	16 (3.25%)
Special Education	17 (3.45%)
Teaching of Religious Culture and Ethics	4 (0.81%)
Turkish Language and Literature Education	3 (0.61%)
Turkish Education	242 (49.09%)
Age (mean ± SD)	21.39 (2.99)

**Table 2:** Factor structure with load values.

Items	Factor 1	Factor 2	Factor 3	Factor 4	Factor 5
I can draw inferences about the text I read. (Item 31)	0.783				
I can determine what the text I read has contributed to me. (Item 32)	0.737				
I can analyze the text in my mind. (Item 34)	0.750				
I can explain the text I read to others. (Item 35)	0.703				
I can discuss the text I read with others. (Item 39)	0.723				
I can shape my future readings based on the text I read. (Item 41)	0.607				
I can connect the text I read with texts I have read previously. (Item 42)	0.652				
I can apply the information I learned from the text to my life. (Item 48)	0.633				
I can interpret how the author’s opinions influenced the text I read. (Item 49)	0.574				
I can underline the parts of the text that catch my attention. (Item 19)		0.845			
I can review the parts I have underlined while reading. (Item 28)		0.919			
I can review the notes I took about the text. (Item 29)		0.703			
I can review the sections I have underlined in the text. (Item 30)		0.920			
I can take precautions to protect my physical health while reading (e.g., resting eyes, sitting upright). (Item 20)			0.781		
I can adjust my reading speed. (Item 21)			0.631		
I can adjust my reading position (e.g., sitting posture, how I hold the book). (Item 23)			0.790		
I can determine the duration of my reading. (Item 24)			0.742		
I can research the creators of the text I am going to read (e.g., author, publisher, translator). (Item 1)				0.764	
I can research the time period in which the text I am going to read was written. (Item 2)				0.795	
I can research the content of the text I am going to read. (Item 3)				0.715	
I can research the purpose for which the text I am going to read was written. (Item 4)				0.687	
I can share my review of the text I read with others. (Item 37)					0.500
I can research the reviews written about the text after reading. (Item 45)					0.768
I can research the comments made about the text after reading. (Item 46)					0.825

**Table 3:** Characteristics and variance contribution rates of factors.

Component	Initial Eigenvalues			Rotation Sums of Squared Loadings		
	Eigenvalue	% of Variance	Cumulative %	Eigenvalue	% of Variance	Cumulative %
Factor 1	7.90	36.93	32.93	4.82	20.10	20.10
Factor 2	2.49	10.38	43.31	3.16	13.16	33.26
Factor 3	1.94	8.07	51.36	2.58	10.76	44.02
Factor 4	1.68	7.01	58.39	2.55	10.64	54.66
Factor 5	1.28	5.32	63.71	2.17	2.17	63.71

**Table 4:** Cronbach’s Alpha values for total scale and subscales.

Factors	Cronbach’s α
Cognitive Strategies	0.895
Note-Taking Strategies	0.906
Exploration and Preparation Strategies	0.775
Physical and Process-Based Strategies	0.791
Reflective and Analytical Strategies	0.708
Total Scale	0.899

**Table 5:** Correlation coefficients for total scale and subscales.

	Factor 1	Factor 2	Factor 3	Factor4	Factor 5	Total
**Factor 1**	1					
**Factor 2**	0.345 *	1				
**Factor 3**	0.445 *	0.271 *	1			
**Factor 4**	0.418 *	0.347 *	0.234 *	1		
**Factor 5**	0.532 *	0.322 *	0.303 *	0.419 *	1	
**Total**	0.814 *	0.683 *	0.635 *	0.660 *	0.691 *	1

* *p* < 0.01 for all correlation coefficients marked with an asterisk (*).

## Data Availability

The data utilized in this study can be provided by the corresponding author upon a justified request.

## Supplementary Materials

The following supporting information can be downloaded at https://www.mdpi.com/article/10.3390/bs15081002/s1: Table S1. Final form of the scale; Table S2. Sample characteristics of qualitative stage; Table S3. Semi-structured interview form; Table S4. English version of qualitative findings with sample references; Table S5. Turkish version of qualitative findings with sample references; Table S6. Content validity ratios of expert review; Table S7. Thematic analysis findings for items that met the content validity ratio threshold; Table S8. Item discrimination results; Table S9. Factor and scale level discrimination results; Table S10. Sample characteristics of confirmatory factory analysis; Table S11. Sample characteristics of nomological network; Figure S1. Scree plot.
