# Supplementary material for: Initial Development and Psychometric Validation of the Self-Efficacy Scale for Informational Reading Strategies in Teacher Candidates
Source: Behav Sci (Basel). 2025 Jul 23;15(8):1002. doi: 10.3390/bs15081002 (PMC12382810; doi:10.3390/bs15081002)
Supplement: Supplementary file 1 [file behavsci-15-01002-s001.zip › behavsci-3717415-supplementary.pdf]

Supplementary Materials

Tables

Table S1. Final form of the scale 2
Table S2. Sample characteristics of qualitative stage 4
Table S3. Semi-structured interview form 5
Table S4. English version of qualitative findings with sample references....................................6
Table S5. Turkish version of qualitative findings with sample references 8
Table S6. Content validity ratios of expert review....................................10
Table S7. Thematic analysis findings for items that met the content validity ratio threshold....................................12
Table S8. Item discrimination results....................................16
Table S9. Factor and scale level discrimination results 18
Table S10. Sample characteristics of confirmatory factory analysis 19
Table S11. Sample characteristics of nomological network 20

Figures

Figure 1. Scree plot....................................21

Table S1. Final form of the scale

English Version of the Form								
**Instructions:** Please indicate how often you believe you can perform the following behaviors using the provided scale:<br>1 = I can never do it<br>2 = I can rarely do it<br>3 = I can sometimes not do it<br>4 = I am undecided<br>5 = I can sometimes do it<br>6 = I can often do it<br>7 = I can always do it								
		1	2	3	4	5	6	7
Cognitive Strategies	1. I can draw inferences about the text I read.							
	2. I can explain the text I read to others.							
	3. I can discuss the text I read with others.							
	4. I can connect the text I read with texts I have read previously.							
	5. I can benefit from the information I learned from the text in my life.							
	6. I can determine what the text I read has contributed to me.							
	7. I can analyze the text in my mind.							
	8. I can shape my future readings based on the text I read.							
	9. I can interpret how the author's opinions influenced the text I read.							
Note-Taking Strategies	10. I can underline the parts of the text that catch my attention.							
	11. I can review the parts I have underlined while reading.							
	12. I can review the notes I took about the text.							
	13. I can review the sections I have underlined in the text.							
Physical and Process-Based Strategies	14. I can take precautions to protect my physical health while reading (e.g., resting eyes, sitting upright).							
	15. I can adjust my reading speed.							
	16. I can adjust my reading position (e.g., sitting posture, how I hold the book).							
	17. I can determine the duration of my reading.							
Exploration and Preparation Strategies	18. I can research the creators of the text I am going to read (e.g., author, publisher, translator).							
	19. I can research the time period in which the text I am going to read was written.							
	20. I can research the content of the text I am going to read.							
	21. I can research the purpose for which the text I am going to read was written.							
Reflective and Analytical Strategies	22. I can share my review of the text I read with others.							
	23. I can research the reviews written about the text after reading.							
	24. I can research the comments made about the text after reading.							

Table S1. (Continue)

Turkish Version of the Form

Talimatlar: Lütfen verilen ölçeği kullanarak aşağıdaki davranışları ne sıklıkla gerçekleştirebileceğinize inandığınızı belirtin:
1 = Hiçbir zaman yapamam
2 = Çoklukla yapamam
3 = Bazen yapamam
4 = Kararsızım
5 = Bazen yapabilirim
6 = Çoklukla yapabilirim
7 = Her zaman yapabilirim

		1	2	3	4	5	6	7
Zihnî Stratejiler	1. Okuduğum metin hakkında çıkarım yapabilirim.							
	2. Okuduğum metnin bana neler kattığını belirleyebilirim.							
	3. Metni zihnimde analiz edebilirim.							
	4. Okuduğum metni başkalarına anlatabilirim.							
	5. Metinden öğrendiğim bilgilerden hayatımda faydalanabilirim.							
	6. Okuduğum metinle daha önce okuduğum metinler arasında ilişkilendirme yapabilirim.							
	7. Okuduğum metni başkalarıyla tartışabilirim.							
	8. Okuduğum metinden yola çıkarak sonraki okumalarımı şekillendirebilirim.							
	9. Yazarın görüşlerinin okuduğum metne olan etkisini yorumlayabilirim.							
Not Alma Stratejileri	10. Metindeki ilgimi çeken kısımların altını çizebilirim.							
	11. Okuma sırasında altını çizdiğim kısımları gözden geçirebilirim.							
	12. Metin hakkında aldığım notları gözden geçirebilirim.							
	13. Metindeki altını çizdiğim yerleri gözden geçirebilirim.							
Fiziki ve Süreç Odaklı Stratejiler	14. Okuma sırasında beden sağlığımı korumak için tedbirler alabilirim. (Gözleri dinlendirme, dik oturma vb.)							
	15. Okuma hızımı ayarlayabilirim.							
	16. Okuma pozisyonumu düzenleyebilirim. (Oturma şekli, kitabı tutuş şekli vb.)							
	17. Okuma süresini belirleyebilirim.							
Keşif ve Hazırlık Stratejileri	18. Okuyacağım bir metni hazırlayanlar hakkında (yazar, yayınevi, çevirmen vb.) araştırma yapabilirim.							
	19. Okuyacağım metnin yazıldığı dönem hakkında araştırma yapabilirim.							
	20. Okuyacağım metnin içeriği hakkında araştırma yapabilirim.							
	21. Okuyacağım metnin yazılış amacı hakkında araştırma yapabilirim.							
Yansıtıcı ve Analitik Stratejiler	22. Okuduğum metnin inceleme yazısını başkalarıyla paylaşabilirim.							
	23. Okuma sonrası metin hakkında yapılan incelemeleri araştırabilirim.							
	24. Okuma sonrası metin hakkında yapılan yorumları araştırabilirim.							

Table S2. Sample characteristics of qualitative stage

Participants	University	Department	Class
1	Yildiz Technical University	Turkish Education	3
2	Yildiz Technical University	Turkish Education	2
3	Yildiz Technical University	Elementary Mathematics Education	4
4	Yildiz Technical University	Social Sciences Education	3
5	Trakya University	Primary Teacher Education	4
6	Yildiz Technical University	Turkish Education	2
7	İstanbul Kültür University	Psychological Counseling and Guidance	4
8	Yildiz Technical University	Turkish Education	4
9	Karadeniz Technical University	Primary Teacher Education	2
10	Yildiz Technical University	Science Education	2
11	Yildiz Technical University	Primary Teacher Education	2
12	Yildiz Technical University	Turkish Education	4
13	Yildiz Technical University	Elementary Mathematics Education	4
14	Yildiz Technical University	Primary Teacher Education	4
15	Yildiz Technical University	Social Sciences Education	4
16	Yildiz Technical University	Elementary Mathematics Education	4
17	Yildiz Technical University	Psychological Counseling and Guidance	3
18	Yildiz Technical University	Elementary Mathematics Education	4
19	Yildiz Technical University	Science Education	4
20	Yildiz Technical University	Primary Teacher Education	2
21	Yildiz Technical University	Turkish Education	4
22	Yildiz Technical University	Turkish Education	4
23	Yildiz Technical University	Primary Teacher Education	3
24	Yildiz Technical University	Turkish Education	4
25	Yildiz Technical University	Primary Teacher Education	4
26	Yildiz Technical University	Turkish Education	3
27	Yildiz Technical University	Turkish Education	4
28	Yildiz Technical University	Turkish Education	4
29	Yildiz Technical University	Primary Teacher Education	1
30	Yildiz Technical University	Elementary Mathematics Education	2
31	Yildiz Technical University	Psychological Counseling and Guidance	1
32	Yildiz Technical University	Social Sciences Education	1
33	Yildiz Technical University	Turkish Education	1

Table S3. Semi-structured interview form

English Version of the Form
This interview form is designed to examine teacher candidates' informational reading strategies across three distinct phases: pre-reading, during-reading, and post-reading. The questions aim to address these strategies within the context of self-efficacy. In this way, the goal is to identify self-efficacy behaviors arising from mastery experiences, observing others' strategies (vicarious experiences), receiving feedback (social persuasion), and emotional or physiological states experienced during the reading process.
**1. Pre-Reading Strategies**
1.1. What strategies do you use to prepare for reading?
1.2. What strategies do you use to enhance your confidence and retention before starting the reading process?
**2. During-Reading Strategies**
2.1. What strategies do you use to make your reading process more efficient and effective?
2.2. What strategies do you use to improve your understanding of the text while reading?
2.3. What strategies do you use to maintain focus and enhance retention during reading?
**3. Post-Reading Strategies**
3.1. What strategies do you use to reflect on and reinforce the material after completing your reading?
3.2. What strategies do you use to ensure the material remains memorable and contributes to your confidence in future reading tasks?
**Turkish Version of the Form**
Bu görüşme formu öğretmen adaylarının bilgilendirici okuma stratejilerini okuma öncesi, okuma sırası ve okuma sonrası şeklinde üç farklı aşamada incelemek için tasarlanmıştır. Sorular bu stratejileri öz-yeterlik kontekstinde ele almayı amaçlamaktadır. Böylece vazife başarısı tecrübeleri (mastery experiences), başkalarının stratejilerini gözlemleme (vicarious experiences), geri bildirim alma (social persuasion) ve okuma sürecindeki duygulu veya fizyolojik vaziyetlerden (physiological and emotional states) kaynaklanarak ortaya çıkan öz yeterlilik davranışlarının belirlenmesi amaçlanmaktadır.
**1. Okuma Öncesindeki Stratejiler**
1.1. Okumaya hazırlık için hangi stratejileri kullanıyorsunuz?
1.2. Akılda kalıcılığı sağlamak için hangi stratejileri kullanıyorsunuz?
**2. Okuma Sırasındaki Stratejiler**
2.1. Okumalarınızı verimli hale getirmek için hangi stratejileri kullanıyorsunuz?
2.2. Okuduğunuzu anlamak için hangi stratejileri kullanıyorsunuz?
2.3. Okumalarınızı kalıcı hale getirmek için hangi stratejileri kullanıyorsunuz?
**3. Okuma Sonrasındaki Stratejiler**
3.1. Okumalarınızı verimli hale getirmek için hangi stratejileri kullanıyorsunuz?
3.2. Akılda kalıcılığı sağlamak için hangi stratejileri kullanıyorsunuz?

Table S4. English version of qualitative findings with sample references

Themes (Self-Efficacy Source)	Category	Participant Response
**Mastery Experiences**	Analyzing Text Structure	"Before reading, I try to understand the structure of the text by examining section headings." (P1)<br>"I focus on key parts to facilitate understanding." (P33)
	Conducting Research	"I research the author, book, publisher, and translator." (P3)<br>"I explore the period when the book was written and other sources from that time." (P14)
	Summarizing Key Points	"I summarize the text in my own words." (P5)<br>"I record the main ideas in short notes." (P6)
	Overcoming Challenges	"I reread parts I don't understand or research them online." (P7)<br>"I discuss complex paragraphs with my classmates." (P8)
	Applying New Knowledge to Life	"I use new vocabulary from the text." (P11)<br>"I apply the knowledge I've gained to my projects." (P10)
	Identifying Themes	"I pay attention to the text's subtext and themes." (P9)<br>"Theme analysis helps me connect more deeply with the story." (P12)
	Taking Notes	"I highlight important parts of the text using colored pens." (P13)<br>"I review my notes after reading." (P4)
**Vicarious Experiences**	Seeking Recommendations	"Books recommended by my friends are usually more enjoyable." (P15)<br>"I select new books based on expert recommendations." (P16)
	Learning from Discussions	"Class discussions help me understand different perspectives of the texts." (P20)<br>"Talking about books with friends is enjoyable and informative." (P18)
	Examining Expert Opinions	"Reading the author's preface helps me understand their perspective." (P19)<br>"I master details by reading text analyses." (P17)

Table S4. (Continue)

**Social Persuasion**	Receiving Feedback from Others	"Feedback from my teacher on how to analyze the text is very helpful." (P21)<br>"Class discussions clarify my thoughts on the text." (P26)
	Peer Support	"My friends encourage me to tackle difficult texts." (P23)<br>"The friends I work with explain parts I don't understand." (P24)
	Guidance from Experts	"The author's introductory notes serve as a guide for me." (P25)<br>"I am drawn to expert comments on book recommendations." (P22)
**Emotional and Physical Situations**	Preparing a Suitable Environment	"I keep my reading space quiet and free from distractions." (P27)<br>"I focus better in my personal space." (P29)
	Managing Reading Anxiety	"I take breaks when I feel overwhelmed." (P28)<br>"When parts confuse me, I switch to a different book." (P30)
	Gaining Confidence through Routine	"I read for an hour every day, and it improves me." (P32)<br>"Having a reading routine makes me feel more organized." (P31)
	Adjusting Posture and Lighting	"I adjust the lighting and noise level according to my reading style." (P33)<br>"I sit upright and read without straining my eyes." (P1)

Table S5. Turkish version of qualitative findings with sample references

**Temalar (Öz-Yeterlik Kaynakları)**	**Kategori**	**Katılımcı Görüşü**
**Başarı Deneyimleri**	Metin Yapısını Çözümleme	"Okuma yapmadan önce metnin yapısını anlamaya çalışıyorum, bölüm başlıklarını inceliyorum." (K1)<br>"Metni anlama kolaylığı için önemli kısımlara odaklanıyorum." (K33)
	Araştırma Yapma	"Yazar, kitap, yayınevi ve çevirmen hakkında araştırma yaparım." (K3)<br>"Kitabın yazıldığı dönemi ve o dönemle ilgili diğer kaynakları incelerim." (K14)
	Önemli Noktaları Özetleme	"Okuduğum metni kendi kelimelerimle özetliyorum." (K5)<br>"Metnin ana fikirlerini kısa notlarla kaydediyorum." (K6)
	Zorlukları Aşma	"Anlamadığım yerleri tekrar okuyorum veya internette araştırıyorum." (K7)<br>"Karmaşık paragrafları sınıf arkadaşlarımla tartışıyorum." (K8)
	Yeni Bilgiyi Hayata Uygulama	"Metinde geçen yeni kelimeleri kullanıyorum." (K11)<br>"Öğrendiğim bilgileri projelerimde uyguluyorum." (K10)
	Temaları Belirleme	"Metnin alt metinlerine ve temalarına dikkat ediyorum." (K9)<br>"Tema analizleri, hikaye ile daha derin bağlar kurmama yardımcı oluyor." (K12)
	Not Alma	"Renkli kalemler kullanarak metnin önemli yerlerini vurguluyorum." (K13)<br>"Okuma sonrası notlarımı tekrar gözden geçiriyorum." (K4)
**Başkalarını Gözlemleme**	Öneriler Arama	"Arkadaşlarımın bana önerdiği kitaplar genelde daha keyifli oluyor." (K15)<br>"Uzman önerilerini okuyarak yeni kitaplar seçiyorum." (K16)
	Tartışmalardan Öğrenme	"Sınıfta yaptığımız tartışmalar, metinlerin daha farklı yönlerini anlamama yardımcı oluyor." (K20)<br>"Arkadaş sohbetlerinde kitap hakkında konuşmak keyifli ve bilgilendirici." (K18)
	Uzman Görüşlerini Gözlemleme	"Yazarın önsözünü okumak, onun bakış açısını anlamama yardımcı oluyor." (K19)<br>"Metin incelemelerini okuyarak detaylara hâkim oluyorum." (K17)

Table S5. (Continue)

**Sözel İkna**	Diğerlerinden Geri Bildirim Alma	"Hocamın bana metni çözümlemek için verdiği geri bildirimler yol gösterici oluyor." (K21)<br>"Sınıf tartışmalarından sonra metin hakkında daha net fikirlerim oluyor." (K26)
	Akran Desteği	"Arkadaşlarım bana zor metinlerin üstesinden gelebileceğimi söylüyor." (K23)<br>"Birlikte çalıştığım arkadaşlar, anlamadığım yerleri açıklıyor." (K24)
	Uzman Yönlendirmesi	"Yazarın başlangıç yazıları benim için rehberlik sağlıyor." (K25)<br>"Metin önerileri üzerine uzman yorumları dikkatimi çekiyor." (K22)
**Duygusal ve Fiziksel Durumlar**	Uygun Ortam Hazırlama	"Okuma alanımı sessiz ve dikkat dağıtıcı şeylerden uzak tutuyorum." (K27)<br>"Kendi alanımda, daha rahat odaklanıyorum." (K29)
	Okuma Kaygısını Yönetme	"Yoğun olduğumda ara veriyorum." (K28)<br>"Anlamadığım kısımlar beni bunalttığında farklı bir kitaba geçiyorum." (K30)
	Rutinle Özgüven Kazanma	"Her gün bir saat okuma yapıyorum ve bu beni geliştiriyor." (K32)<br>"Okuma rutini sayesinde daha organize hissediyorum." (K31)
	Duruş ve Işık Ayarlama	"Okuma tarzıma göre ışık ve ses seviyesini ayarlıyorum." (P33)<br>"Dik oturur ve gözlerimi yormadan okurum." (P1)

Table S6. Content validity ratios of expert review

	Expert 1	Expert 2	Expert 3	Expert 4	Expert 5	Expert 6	Expert 7	Expert 8	Expert 9	CVR
**Item 1**	x	x	x	x	x	x	x		x	.78
**Item 2**	x	x	x	x		x		X	x	.56
**Item 3**	x	x	x		x		x	X	x	.56
**Item 4**	x	x	x	x	x	x	x		x	.78
**Item 5**	x	x	x	x	x	x			x	.56
**Item 6**	x	x	x	x	x	x			x	.56
**Item 7**	x	x	x	x	x		x	x	x	.78
**Item 8**	x	x	x	x	x		x	x	x	.78
**Item 9**	x	x	x	x	x	x	x			.56
**Item 10**	x	x	x	x	x	x	x			.56
**Item 11**	x	x				x	x	x	x	.33
**Item 12**	x	x	x	x	x			x	x	.56
**Item 13**			x		x		x	x	x	.11
**Item 14**	x		x	x	x	x	x	x	x	.78
**Item 15**		x	x	x	x			x	x	.33
**Item 16**	x	x	x	x		x	x	x	x	.78
**Item 17**			x	x					x	-.33
**Item 18**	x	x	x	x	x	x			x	.56
**Item 19**	x	x	x	x	x	x	x	x	x	1
**Item 20**	x	x	x	x	x	x	x			.56
**Item 21**			x		x	x	x		x	.11
**Item 22**	x	x	x	x	x	x			x	.56
**Item 23**	x	x	x	x	x	x		x	x	.78
**Item 24**	x	x	x	x	x		x	x	x	.78
**Item 25**	x	x		x	x	x	x	x	x	.78
**Item 26**	x	x	x	x	x	x	x	x	x	1
**Item 27**		x	x		x	x	x		x	.33
**Item 28**	x	x	x	x	x	x	x	x	x	1
**Item 29**	x	x		x	x	x	x	x	x	.78
**Item 30**		x	x		x	x		x	x	.33
**Item 31**	x	x	x	x	x	x	x		x	.78
**Item 32**	x	x	x	x	x	x	x	x	x	1
**Item 33**	x	x		x		x	x		x	.33
**Item 34**	x	x	x	x	x	x	x		x	.78
**Item 35**	x	x	x	x	x	x	x	x	x	1
**Item 36**	x			x	x		x		x	.111
**Item 37**	x	x	x	x	x	x	x	x	x	1
**Item 38**	x	x	x	x	x	x		x	x	.78
**Item 39**	x	x	x	x	x		x	x	x	.78
**Item 40**	x	x	x	x	x	x	x	x	x	1
**Item 41**	x	x	x	x	x	x	x	x	x	1
**Item 42**	x	x	x	x	x	x	x	x	x	1
**Item 43**	x	x	x	x	x	x	x		x	.78
**Item 44**	x	x	x	x	x	x	x		x	.78
**Item 45**	x	x	x	x	x	x	x	x	x	1
**Item 46**	x	x	x		x	x	x	x	x	.78
**Item 47**	x	x	x		x	x	x	x	x	.78
**Item 48**	x	x		x	x	x	x	x	x	.78
**Item 49**	x	x	x	x	x	x	x	x	x	1
**Item 50**	x			x	x	x			x	.11
**Item 51**	x	x	x	x	x	x	x	x		.78
**Item 52**	x	x	x	x	x	x	x	x	x	1

Table S6. (Continue)

**Item 53**	x	x	x	x	x	x	x	x	x	1
**Item 54**	x	x	x	x	x	x	x	x	x	1
**Item 55**	x	x	x	x	x	x		x	x	.78
**Item 56**	x	x	x	x	x	x		x	x	.78
**Item 57**	x	x	x	x	x	x	x	x	x	1
**Item 58**	x	x	x	x	x	x	x	x	x	1
**Item 59**	x	x	x	x	x	x	x	x	x	1
**Item 60**	x	x	x	x	x	x	x	x	x	1
**Item 61**	x	x	x	x	x	x	x	x	x	1
**Item 62**	x	x	x	x	x	x	x	x	x	1
**Item 63**	x	x	x	x	x	x	x	x	x	1
**Item 64**	x	x	x	x	x	x	x	x	x	1
**Item 65**	x	x	x	x	x	x	x	x	x	1
**Item 66**	x	x	x	x	x	x	x	x	x	1
**Item 67**	x	x	x	x	x	x	x	x	x	1
**Item 68**	x	x	x	x	x	x	x	x	x	1
**Item 69**	x	x	x	x	x	x	x		x	.78
**CVR(Critical) for a panel size (N) of 9 is 1.**										.75

Table S7. Thematic analysis findings for items that met the content validity ratio threshold

Factor	Item (Turkish)	Item (English)
**Cognitive Strategies [=Zihnî Stratejiler]**	31. Okuduğum metin hakkında çıkarım yapabilirim.	I can draw inferences about the text I read.
	32. Okuduğum metnin bana neler kattığını belirleyebilirim.	I can identify what the text I read has contributed to me.
	34. Metni zihnimde analiz edebilirim.	I can analyze the text in my mind.
	35. Okuduğum metni başkalarına anlatabilirim.	I can explain the text I read to others.
	39. Okuduğum metni başkalarıyla tartışabilirim.	I can discuss the text I read with others.
	41. Okuduğum metinden yola çıkarak sonraki okumalarımı şekillendirebilirim.	I can shape my future readings based on the text I read.
	42. Okuduğum metinle daha önce okuduğum metinler arasında ilişkilendirme yapabilirim.	I can connect the text I read with texts I have read previously.
	49. Okuduğum metnin özetini çıkarabilirim.	I can summarize the text I read.
	50. Metinden öğrendiğim yeni kelimeleri hayatımda kullanabilirim.	I can use the new words I learned from the text in my daily life.
	51. Metinden öğrendiğim bilgilerden hayatımda faydalanabilirim.	I can benefit from the information I learned from the text in my life.
	52. Yazarın görüşlerinin okuduğum metne olan etkisini yorumlayabilirim.	I can interpret how the author's opinions influenced the text I read.

Table S7. (Continue)

**Note-Taking Strategies**	16. Metinde mühim gördüğüm yerleri not alabilirim.	I can take notes on the parts of the text I see important.
	17. Metindeki ilgimi çeken yerleri not alabilirim.	I can take notes on the parts of the text that catch my attention.
	19. Metindeki ilgimi çeken kısımların altını çizebilirim.	I can underline the parts of the text that catch my attention.
	27. Okuma sırasında not aldığım kısımları gözden geçirebilirim.	I can review the parts I took notes on during reading.
	28. Okuma sırasında altını çizdiğim kısımları gözden geçirebilirim.	I can review the parts I underlined during reading.
	29. Metin hakkında aldığım notları gözden geçirebilirim.	I can review the notes I took about the text.
	30. Metindeki altını çizdiğim yerleri gözden geçirebilirim.	I can review the sections I have underlined in the text.
**Exploration and Preparation Strategies [=Araştırma ve Hazırlık Stratejileri]**	1. Okuyacağım bir metni hazırlayanlar hakkında araştırma yapabilirim.	I can research the creators of the text I am going to read (e.g., author, publisher, translator).
	2. Okuyacağım metnin yazıldığı dönem hakkında araştırma yapabilirim.	I can research the time period in which the text I am going to read was written.
	3. Okuyacağım metnin içeriği hakkında araştırma yapabilirim.	I can research the content of the text I am going to read.
	4. Okuyacağım metnin yazılış amacı hakkında araştırma yapabilirim.	I can research the purpose for which the text I am going to read was written.
	5. Okuyacağım metnin türü hakkında araştırma yapabilirim.	I can research the genre of the text I am going to read.
	7. Okuyacağım metnin formatını belirleyebilirim.	I can determine the format of the text I am going to read (print or digital).

Table S7. (Continue)

	8. Okuyacağım metnin konusunu belirleyebilirim.	I can determine the topic of the text I am going to read.
	9. Okuma amacıma yönelik metin seçebilirim.	I can select a text according to my reading purpose.
	10. Metnin başlığından okuyacağım metin hakkında ön bilgi sahibi olabilirim.	I can gain prior knowledge about the text I am going to read by examining the title.
	11. Okuma amacımı belirleyebilirim.	I can determine my reading purpose.
	12. Okuma sürecini önceden tasarlayabilirim.	I can plan the reading process in advance.
	15. Metnin sonuna yönelik tahminlerde bulunabilirim.	I can make predictions about the ending of the text.
	48. Okuma sonrasında metnin yazarı hakkında araştırma yapabilirim.	I can research the author of the text after reading.
**Physical and Process-Based Strategies [=Fiziki ve Süreç Odaklı Stratejiler]**	20. Okuma sırasında beden sağlığımı korumak için tedbirler alabilirim.	I can take measures to protect my physical health while reading (e.g., resting my eyes, sitting upright).
	21. Okuma hızımı ayarlayabilirim.	I can adjust my reading speed.
	23. Okuma pozisyonumu düzenleyebilirim. (Oturma şekli, kitabı tutuş şekli vb.)	I can adjust my reading position (e.g., sitting posture, how I hold the book).
	24. Okuma süresini belirleyebilirim.	I can determine the duration of my reading.
	25. Okuma zamanımı düzenleyebilirim.	I can schedule my reading time.
	26. Metni sesli/sessiz okuyabilirim.	I can read aloud or silently.

Table S7. (Continue)

	33. Metnin hedef kitleye uygun olup olmadığını belirleyebilirim.	I can determine whether the text is appropriate for the target audience.
	36. Okuduğum metin hakkında inceleme yazısı yazabilirim.	I can write a review about the text I read.
	37. Okuduğum metnin inceleme yazısını başkalarıyla paylaşabilirim.	I can share the review I wrote about the text with others.
	38. Metni okurken aldığım notları ve alıntıları başkalarıyla paylaşabilirim.	I can share the notes and quotations I took while reading with others.
**Reflective and Analytical Strategies [=Yansıtıcı ve Analitik Stratejiler]**	40. Okuduğum metindeki fikirleri eleştirebilirim.	I can critique the ideas in the text I read.
	43. Okuma sonrası araştırma yapabilirim.	I can conduct research after reading.
	44. Okuma sırasında anlamını çıkaramadığım kelimeleri araştırabilirim.	I can research unfamiliar words I cannot interpret during reading.
	45. Okuma sonrası metin hakkında yapılan incelemeleri araştırabilirim.	I can research the reviews written about the text after reading.
	46. Okuma sonrası metin hakkında yapılan yorumları araştırabilirim.	I can research the comments made about the text after reading.
	47. Okuma sonrası metinde geçen bilgileri araştırabilirim.	I can research the information mentioned in the text after reading.

Table S8. Item discrimination results

Item	Groups	*N*	$\bar{x}$	*ss*	*Sh* $\bar{x}$	*t Test* *t*	*Sd*	*p*
Item 31	Upper	134	7.00	.000	.000	21.431	266	< .001
	Lower	134	5.24	.95	.08			
Item 35	Upper	134	6.65	.58	.05	11.313	266	< .001
	Lower	134	5.44	1.09	.09			
Item 39	Upper	134	7.00	.000	.000	32.388	266	< .001
	Lower	134	4.63	.846	.846			
Item 42	Upper	134	7.00	.000	.000	31.515	266	< .001
	Lower	134	4.46	.96	.08			
Item 51	Upper	134	7.00	.000	.000	33.046	266	< .001
	Lower	134	4.60	.84	.07			
Item 32	Upper	134	7.00	.000	.000	19.860	266	< .001
	Lower	134	5.09	1.11	.10			
Item 34	Upper	134	7.00	.000	.000	24.741	266	< .001
	Lower	134	4.94	.96	.08			
Item 41	Upper	134	7.00	.000	.000	34.230	266	< .001
	Lower	134	4.55	.83	.07			
Item 52	Upper	134	6.96	.19	.02	34.543	266	< .001
	Lower	134	4.37	.85	.07			
Item 28	Upper	134	7.00	.000	.000	28.100	266	< .001
	Lower	134	3.19	1.57	.14			
Item 30	Upper	134	7.00	.000	.000	27.117	266	< .001
	Lower	134	3.30	1.58	.14			
Item 19	Upper	134	7.00	.000	.000	25.417	266	< .001
	Lower	134	3.23	1.72	.15			
Item 29	Upper	134	7.00	.000	.000	27.857	266	< .001
	Lower	134	3.44	1.48	.13			
Item 2	Upper	134	6.40	.49	.04	26.480	266	< .001
	Lower	134	3.03	1.39	.12			
Item 1	Upper	134	6.51	.50	.04	23.410	266	< .001
	Lower	134	3.66	1.32	.11			
Item 3	Upper	134	7.00	.000	.000	27.756	266	< .001
	Lower	134	4.39	1.09	.09			
Item 4	Upper	134	6.60	.49	.04	26.285	266	< .001
	Lower	134	3.47	1.29	.11			
Item 23	Upper	134	7.00	.000	.000	28580	266	< .001
	Lower	134	3.77	1.31	.11			
Item 20	Upper	134	6.52	.50	.04	41.418	266	< .001
	Lower	134	2.60	.97	.08			
Item 24	Upper	134	6.89	.32	.03	32.910	266	< .001
	Lower	134	3.04	1.31	.11			

Table S8. (Continue)

Item 21	Upper	134	6.96	.21	.02	31.690	266	< .001
	Lower	134	3.87	1.12	.10			
Item 46	Upper	134	6.98	.15	.01	29.231	266	< .001
	Lower	134	3.74	1.27	.11			
Item 45	Upper	134	6.73	.45	.04	32.052	266	< .001
	Lower	134	3.78	1.166	.10			
Item 37	Upper	134	6.57	.50	.04	36.269	266	< .001
	Lower	134	2.64	1.15	.10			

Table S9. Factor and scale level discrimination results

Factor 1	Upper	134	6.79	.18	.02	30.580	266	< .001
	Lower	134	5.06	.63	.05			
Factor 2	Upper	134	6.86	.17	.01	29.445	266	< .001
	Lower	134	3.49	1.31	.11			
Factor 3	Upper	134	6.58	.35	.03	36.566	266	< .001
	Lower	134	3.77	.82	.07			
Factor 4	Upper	134	6.45	.37	.03	34.285	266	< .001
	Lower	134	3.92	.77	.07			
Factor 5	Upper	134	6.49	.40	.03	35.260	266	< .001
	Lower	134	3.59	.86	.07			
Scale Total	Upper	134	6.41	.27	.02	32.906	266	< .001
	Lower	134	4.66	.55	.05			

Table S10. Sample characteristics of confirmatory factory analysis

Variable	n= 388 (%)
Sex	
Man	90 (23.20%)
Woman	298 (76.80%)
Undergraduate program	
Computer Education and Instructional Technology	5 (1.28%)
English Language Teaching	72 (18.55%)
Mathematics Education	21 (5.41%)
Preschool Education	56 (14.43%)
Primary Teacher Education	20 (5.15%)
Science Education	31 (7.99%)
Social Studies Education	5 (1.28%)
Turkish Education	178 (45.88%)
Age (mean ± SD)	20.95 (2.57)

Table S11. Sample characteristics of nomological network

Variable	n= 294 (%)
Sex	
Man	63 (21.4%)
Woman	231 (78.6%)
Undergraduate program	
Computer Education and Instructional Technology	6 (2%)
English Language Teaching	35 (11.9%)
Mathematics Education	36 (12.2%)
Preschool Education	36 (12.2%)
Primary Teacher Education	28 (9.5%)
Psychological Counseling and Guidance	6 (2%)
Science Education	41 (13.99%)
Social Studies Education	12 (4.1%)
Turkish Education	94 (32%)
Age (mean ± SD)	21.46 (2.29)

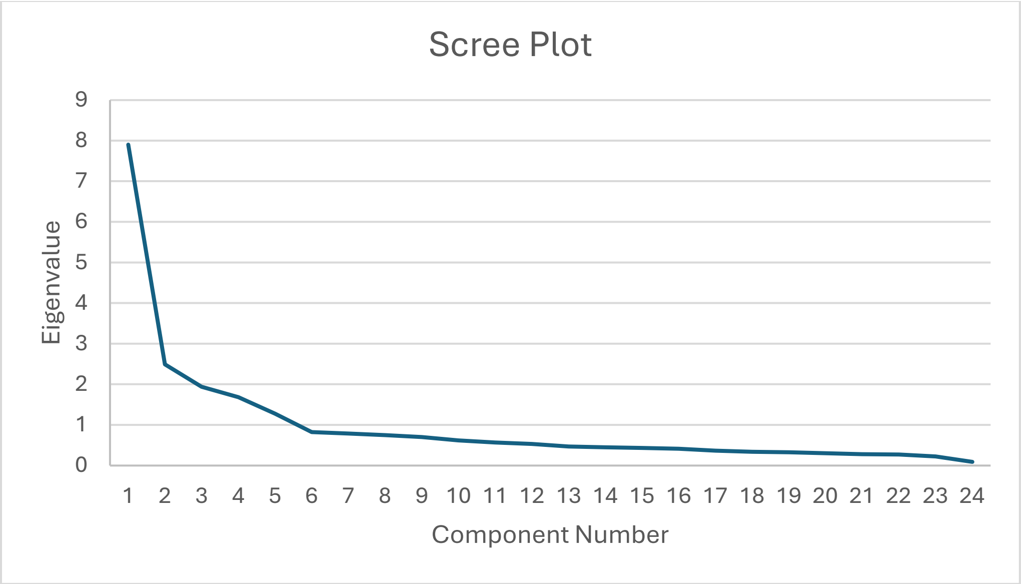

Figure S1. Scree plot.
